# The Dietary Supplement *γ*-Oryzanol Attenuates Hepatic Ischemia Reperfusion Injury via Inhibiting Endoplasmic Reticulum Stress and HMGB1/NLRP3 Inflammasome

**DOI:** 10.1155/2021/4628050

**Published:** 2021-09-02

**Authors:** Yichao Du, Furui Zhong, Huanli Cheng, Tongxi Li, Yifan Chen, Peng Tan, Meizhou Huang, Tiancheng Liang, Yu Liu, Xianming Xia, Wenguang Fu

**Affiliations:** ^1^Academician (Expert) Workstation of Sichuan Province, The Affiliated Hospital of Southwest Medical University, Luzhou 646000, China; ^2^Department of Hepatobiliary Surgery, The Affiliated Hospital of Southwest Medical University, Luzhou 646000, China; ^3^Department of General Surgery, Zigong Fourth People's Hospital, Zigong 643000, China; ^4^Luzhou Municipal Hospital of Traditional Chinese Medicine, Luzhou 646000, China; ^5^Xichang People's Hospital, Xichang 615000, China; ^6^Nuclear Medicine and Molecular Imaging Key Laboratory of Sichuan Province, Luzhou 646000, China

## Abstract

The purpose of this study is to investigate the protective effect of *γ*-oryzanol (ORY) against hepatic ischemia reperfusion (HIR) injury and the potential protective mechanisms of ORY. ORY is an important biologically active ingredient isolated from rice bran oil, which has anti-inflammatory and antiapoptotic effects. However, it is still unknown whether ORY can protect the liver from the HIR damage. In this study, ORY was administered orally for seven days, after which the animals were subjected to liver ischemia for 60 minutes and reperfused for 6 hours. Related indicators were analyzed. The results showed that ORY pretreatment significantly reduced the levels of AST and ALT, relieved hepatocellular damage and apoptosis, and attenuated the exhaustion of SOD and GSH and accumulation of MDA and MPO. Interestingly, ORY treatment could significantly decreased ER stress. Furthermore, ORY pretreatment remarkably reduced the protein expressions of HMGB1, NLRP3, caspase-1 (p20), and IL-1*β* to protect the liver from I/R-induced inflammasome activation and apoptosis. In conclusion, we demonstrated the potential effect of ORY in modulating oxidative stress, endoplasmic reticulum stress, and inflammasome activation during HIR.

## 1. Introduction

Hepatic ischemia reperfusion (HIR) is a physiological and pathological phenomenon which can hardly be avoided in certain types of surgeries and associated with liver injury, liver transplantation, and hepatectomy [1]. HIR injury (HIRI) is based on dynamic interrelated events, including hypoxic stress and energy deficiency induced by ischemia in the prophase and severe inflammation induced by reperfusion in the later phase [2]. However, there is still a lack of effective methods to improve HIRI [3]. Therefore, HIRI must be given considerable attention.

In recent years, it has been found that endoplasmic reticulum (ER) stress plays an important role in early HIRI [4]. Hypoxia and ATP deficiency, calcium overload, reactive oxygen species (ROS), and other factors can trigger ER stress response [5]. In order to restore normal ER function to solve organelle dysfunction, the unfolded protein response (UPR) is activated [6]. UPR is caused by the increase of intra-ER chaperone glucose-regulated protein 78 (GRP78) and its dissociation with activating transcription factor 6 (ATF6), inositol requiring enzyme 1 (IRE1), and protein kinase R-like endoplasmic reticulum kinase (PERK) [7]. However, if cells fail to resolve ER stress or the damage continues to increase, cell death may follow.

The highly mobile base box 1 (HMGB1) is a highly conserved nuclear protein that will be released into the extracellular environment in the form of damage-related molecular patterns (DAMPs) when the cell is under stress or damaged [8]. Previous studies have shown that DAMPs act through Toll-like receptor 2 (TLR2), TLR4, or TLR9 and advanced glycosylation end-product receptors (RAGE), activating NOD (nucleoside) containing 3 (NLRP3) inflammasomes Acid binds to the oligomerization domain-like receptor family, which in turn promotes the release of IL-1*β*, hepatocyte necrosis, and aseptic inflammatory response to the innate immune system [9, 10]. DAMPs are released by necrotic hepatocyte during the inflammatory response stage to cause inflammation, which in turn causes new cell lysis and DAMP release, thus repeatedly stimulating inflammation and eventually amplifying the inflammatory cascade [11]. Therefore, inhibition of the ER stress pathway and inflammatory response may provide a potentially therapeutic intervention approach for HIRI.

**Figure 1:**
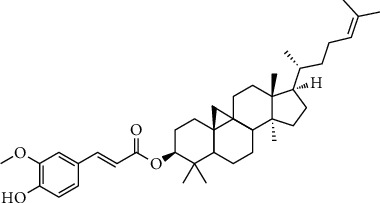
Chemical structure of *γ*-oryzanol. The molecular formula of *γ*-oryzanol (C_40_H_58_O_4_).

For the past few years, increasing attention has been focused on the utilization of bioactive component derived from food components in the prevention of liver diseases [12–14]. *γ*-Oryzanol (ORY, Figure 1) is an essential bioactive component isolated from rice bran oil [15]. Nowadays, ORY has been shown to play a protective role in various models of liver disease by exerting maintenance of metabolic homeostasis, anti-inflammatory, antitumor, and antioxidative effects [16–18]. However, its effects on ER stress and HMGB1/NLRP3-mediated inflammatory response are unclear. Thus, we used oral ORY supplementation as a preventive agent against HIRI and explore the underlying molecular mechanisms in the present study.

## 2. Materials and Methods

### 2.1. Materials

ORY was purchased from Shanghai Macklin Biochemical Co., Ltd. (purity above 99%, Shanghai, China). ORY was suspended in a 0.5% carboxymethylcellulose (CMC-Na) distilled water solution. All other chemicals were of analytical grade.

### 2.2. Experimental Protocol

Male C57BL/6 mice (8–10 weeks old) were bought from Chengdu Dashuo Biotechnological Company (Chengdu, China) and housed in the specific pathogen-free (SPF) facility. After one week of adaptive feeding, mice were randomly divided into the following five groups (10 mice each):

- Group 1 (sham): subjected to sham operation and received 0.5% carboxymethylcellulose (CMC-Na) distilled water solution for 7 days
- Group 2 (I/R): subjected to ischemia/reperfusion (I/R) with gavage of 0.5% carboxymethylcellulose (CMC-Na) distilled water solution (I/R) for seven days before I/R
- Group 3 (I/R+ORY10): subjected to I/R with gavage of 10 mg/kg ORY for 7 days before I/R
- Group 4 (I/R+ORY20): subjected to I/R with gavage of 20 mg/kg ORY for seven days before I/R
- Group 5 (I/R+ORY40): subjected to I/R with gavage of 40 mg/kg ORY for seven days before I/R

Mice were fasted (5–8 hours) and anesthetized with sodium pentobarbital (40 mg/kg, i.p.) and xylazine (10 mg/kg i.p.). Abdominal cavity was opened to expose the hepatic pedicles of the left and middle lobes of the liver. The portal vein and hepatic artery of the middle and left lobes were clamped, causing approximately 70% hepatic ischemia. The ischemia was maintained for sixty minutes, followed by six hours of reperfusion, as previously described in our previous study [19]. Mice were sacrificed to collect liver samples and serum for subsequent examination.

All the experiments were performed in compliance with the Animal Care and Use Committee and Ethics Committee of Southwest Medical University (approval number: 202003-26).

### 2.3. Blood Biochemical Analyses

All blood samples were obtained by cardiac puncture and kept at room temperature for 1 hour and then centrifuged at 4000 rpm for 5 min at 4°C. Serum samples were separated and stored at −80°C. The serum ALT and AST activities were determined according to the method described by Bergmeyer et al. [20, 21]. These Serum biochemical indicators were measured using a commercially available colorimetric assay kit (Jiancheng Biotechnology, Nanjing, China).

### 2.4. Determination of Hepatic GSH, MDA, and SOD Levels

Liver tissue specimens were homogenized in cold saline (1 : 9, *w*/*v*) before the centrifugation at 10000 g for 10 min at 4°C. The supernatants of the hepatic homogenates were collected to measure the levels of MDA, GSH, SOD, and MPO by commercial assay kits, according to the method of [22–25].

### 2.5. Western Blot

Western blot analysis was performed as described in our previous study [26]. Total proteins were extracted from liver tissues and cells by RIPA Lysis Buffer (Beyotime Biotechnology, Shanghai, China) according to the provider's specification. The protein concentration was measured using a BCA assay kits (Beyotime Biotechnology, Shanghai, China). In short, the protein samples were separated by SDS-PAGE and then transferred to 0.22-micron PVDF membranes. The membranes were blocked using 5% nonfat dry milk-TBST buffer for 2 h at room temperature; then, it was incubated overnight at 4°C with primary antibody against Bcl-2 (1 : 1000, #12789-1-AP), Bax (1 : 1000, #50599-2-Ig), HMGB1 (1 : 1000, #66525-1-Ig), NLRP3 (1 : 500, #19771-1-AP), GRP78 (1 : 3000, #11587-1-AP), PERK (1 : 2000, #20582-1-AP), EIF2S1 (1 : 1000, #11170-1-AP), p-EIF2S1 (1 : 1000, #28740-1-AP), CHOP (1 : 1000, #15204-1-AP), *β*-actin (1 : 5000, #66009-1-Ig), and GAPDH (1 : 5000, #60004-1-Ig)—all from Proteintech, Wuhan, China, and IL-1*β* (1 : 1000, #AF5103),p-PERK (1 : 1000, # DF7576), and Cleaved-Caspase 1 (1 : 1000, #AF4005)—all from Affinity Biosciences, China. After being washed with TBST buffer, the membranes were incubated for 1 h with horseradish peroxidase- (HRP-) conjugated secondary antibodies (Protenintech, Wuhan, China). The membranes were visualized using a BeyoECL Moon kit (Beyotime Biotechnology, Shanghai, China). ImageJ software (NIH, USA) was used to quantify the grayscale value of straps. The density values of each sample were normalized against *β*-actin or GAPDH.

### 2.6. Liver Histology and Immunohistochemistry

Liver tissue specimens were fixed in 4% paraformaldehyde for 48 h, then embedded in paraffin, and cut into 5 *μ*m sections. Hematoxylin and eosin (H&E) staining for histological changes and immunohistochemical (IHC) staining for GRP78 (Protenintech, Wuhan, China) were performed in paraffin sections as described previously [27].

### 2.7. Terminal-Deoxynucleotidyl Transferase-Mediated Nick End Labeling Analysis

The paraffin samples were cut into 5 *μ*m sections, followed by dewaxing and hydration. The apoptosis in liver tissue was then analyzed using a terminal-deoxynucleotidyl transferase-mediated nick end labeling (TUNEL) kit (Beyotime, Shanghai, China) according to the manufacturer's instructions as described previously [28].

### 2.8. Cell Culture and Treatment

The mouse hepatocyte cell line AML12 was treated with CoCl_2_ (Sigma-Aldrich, St. Louis, USA) to establish the cell hypoxia model as described previously [29]. AML12 cells were cultured in DMEM/F12 (Hyclone, Logan, USA) containing 10% fetal bovine serum (FBS), ITS liquid media supplement (Sigma-Aldrich, St. Louis, USA), and 40 ng/mL dexamethasone (Solarbio, Beijing, China) at 37°C with 5% CO_2_. The cells divided into four treatment groups. These were the control, ORY (240 *μ*g/mL), CoCl_2_ (300 *μ*M), and CoCl_2_+ORY (240 *μ*g/mL ORY was added at the same time with CoCl_2_ (300 *μ*M)) groups [30]. Levels of ROS in AML12 were determined by DCFH-DA ROS assay kit (Beyotime, Shanghai, China) according to the manufacturer's instruction. The mouse hepatocyte cell line AML12 was treated with tunicamycin (TM) (Solarbio, Beijing, China) as described previously [31]. These were the control, ORY (240 *μ*g/mL), TM (10 *μ*g/mL), and TM+ORY. The cells were pretreated with *γ*-oryzanol for 12 h, followed by treatment with 10 *μ*g/mL tunicamycin for an additional 24 h.

### 2.9. Reactive Oxygen Species (ROS) Production Assay

ROS production was detected by the ROS detection kit (Beyotime, Shanghai, China). In the cell experiments, AML12 were incubated with 10 *μ*m DCFH-DA at 37°C for 30 min. Then, the medium was discarded, the cells were washed in the dark with cold PBS, and the production of ROS was evaluated with fluorescence intensity measured by fluorescence spectroscopy, and images were obtained on a fluorescence microscope (Olympus, Japan).

### 2.10. Statistical Analysis

Statistical analysis was performed with statistical software GraphPad Prism 8.0 (GraphPad 8.0, La Jolla, CA). Data were expressed as mean ± standard deviation (SD). Differences between the experimental groups were compared by one-way ANOVA and Dunnett's multiple comparison test. A *P* value less than 0.05 (*P* < 0.05) was considered significant.

## 3. Results

### 3.1. ORY Alleviated Liver Injury Induced by I/R in Mice

**Figure 2:**
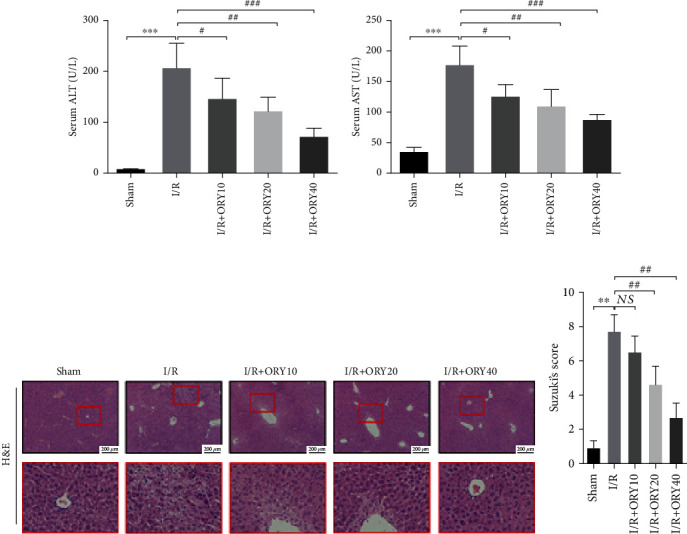
ORY alleviated liver injury induced by I/R in mice. Effect of ORY on the levels of ALT (a) and AST (b) in the serum. (c) Histological evaluation of the liver tissue specimens was conducted by H&E staining (original magnification ×100). (d) Histological severity of I/R-induced hepatic injury was graded using Suzuki's score. Data were expressed as mean ± standard deviation (SD) values (*n* = 6). ^∗^*P* < 0.05, ^∗∗^*P* < 0.01, and ^∗∗∗^*P* < 0.001 versus the sham group; ^#^*P* < 0.05, ^##^*P* < 0.01, and ^###^*P* < 0.001 versus the I/R group; NS: no significance.

As shown in Figures 2(a) and 2(b). Compared with the sham group, the serum levels of AST and ALT were significantly elevated in the I/R groups (*P* < 0.01). Furthermore, compared with the I/R group, ORY pretreatment at different doses (10, 20, and 40 mg/kg) significantly reduced the serum levels of AST and ALT with the most significant reduction being in the I/R+ORY40 group (*P* < 0.01). Morphological and histopathological examinations were evaluated by H&E staining. In the sham group, the liver had normal morphological characteristics of the hepatocytes and complete hepatic lobules. On the contrary, the histopathological manifestations in the I/R group included collapses in the hepatic lobular structure, hemorrhagic foci, and hepatocyte necrosis. However, the ORY pretreatment groups only showed mild degeneration, and the morphology of liver cell nuclei and liver lobules was basically normal. (Figures 2(c) and 2(d)).

### 3.2. ORY Reduced GSH, SOD, MDA, and MPO Levels during HIRI

**Figure 3:**
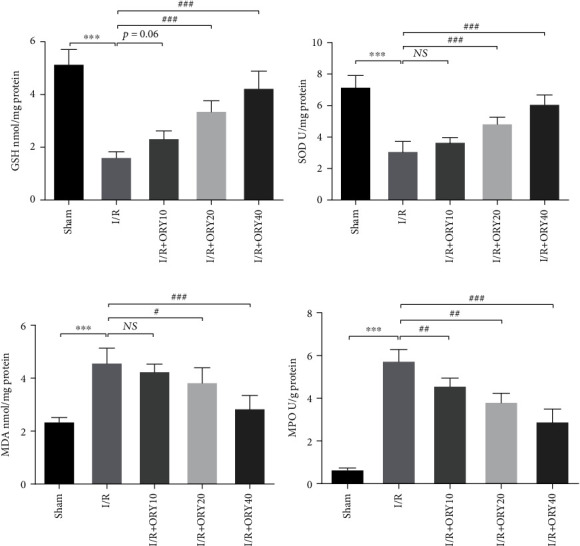
ORY reduced GSH, SOD, MDA, and MPO levels during HIRI. The hepatic tissue GSH concentration (a), SOD activity (b), MDA concentration (c), and MPO activity (d) were assayed by using the corresponding kits. Data are expressed by mean ± standard deviation (SD) values (*n* = 5). ^∗^*P* < 0.05, ^∗∗^*P* < 0.01, and ^∗∗∗^*P* < 0.001 versus the sham group; ^#^*P* < 0.05, ^##^*P* < 0.01, and ^###^*P* < 0.001 versus the I/R group; NS: no significance.

In order to specify the effect of ORY on oxidative stress, lipid peroxidation, and neutrophil infiltration induced by I/R in mice, we first examined the levels of GSH, SOD, MDA, and MPO through commercial kits. The results are presented in Figure 3. In short, compared with the sham group, the contents of antioxidant-related factors GSH and SOD were markedly decreased while the contents of peroxide-related factors which contained MDA and MPO were markedly increased in the I/R group. Furthermore, compared with the I/R group, ORY pretreatment at doses (20 and 40 mg/kg) significantly reduced MDA and MPO contents and enhanced GSH and SOD contents (*P* < 0.01), but the role of ORY 10 mg/kg to improve I/R is less obvious. Therefore, the sham, I/R, I/R+ORY20, and I/R+ORY40 groups were selected for follow-up experiments.

### 3.3. ORY Suppressed Endoplasmic Reticulum Stress during HIRI

**Figure 4:**
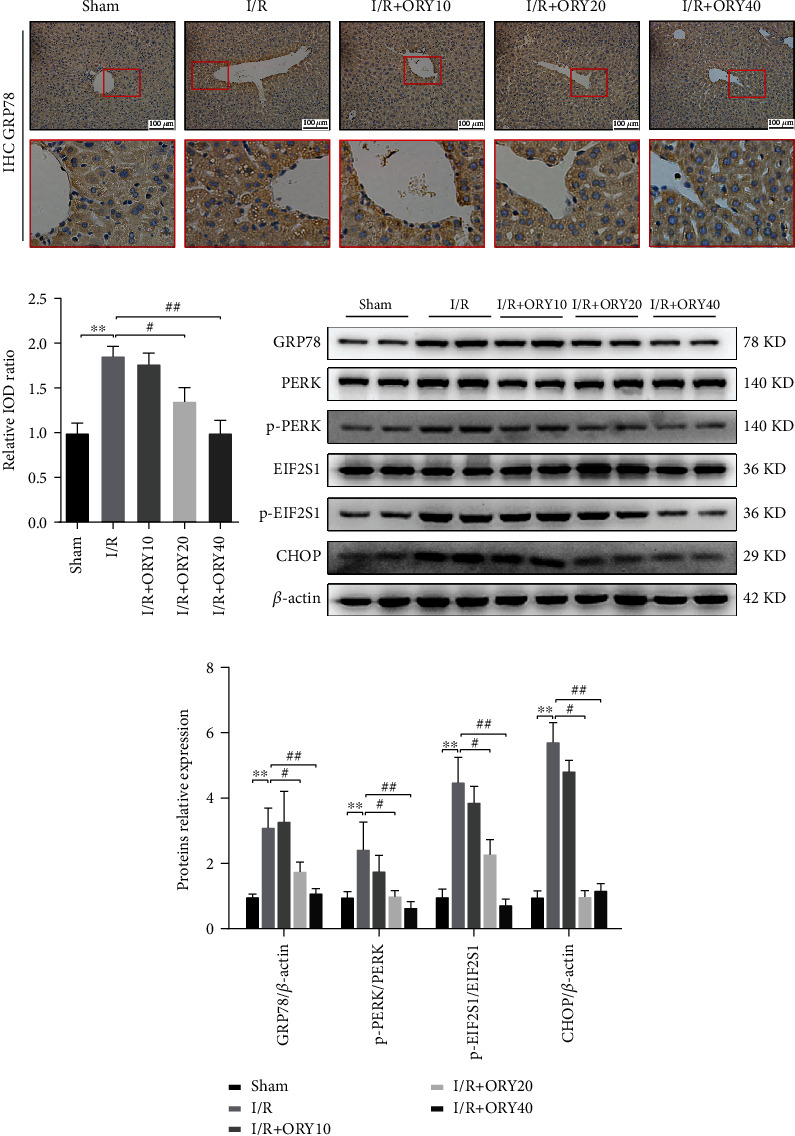
ORY suppressed endoplasmic reticulum stress during HIRI. (a, b) Immunohistochemistry analysis of GRP78 (original magnification ×200). (c, d) Proteins of hepatic tissues were determined by western blotting for the determination of GRP78, p-PERK, p-EIF2S1, and CHOP and *β*-actin expression. Relative protein abundance was semiquantified by densitometry (*n* = 4). Data were expressed as mean ± standard deviation (SD) values. ^∗^*P* < 0.05, ^∗∗^*P* < 0.01, and ^∗∗∗^*P* < 0.001 versus the sham group; ^#^*P* < 0.05, ^##^*P* < 0.01, and ^###^*P* < 0.001 versus the I/R group; NS: no significance.

HIR has been shown to induce ERS and activation of UPR [32, 33]. So, we further analyzed the effect of ORY preconditioning on ER stress by IHC and western blot (Figure 4). IHC analysis of the liver specimen showed that the level of GRP78 protein of the I/R group was significantly higher than that of the sham and ORY (20 and 40 mg/kg) pretreatment groups. Western blot revealed that I/R significantly induced the expression of GRP78, p-PERK, p-EIF2S1, and CHOP, which were all inhibited by ORY.

### 3.4. ORY Attenuated HMGB1/NLRP3 Inflammasome during HIRI

**Figure 5:**
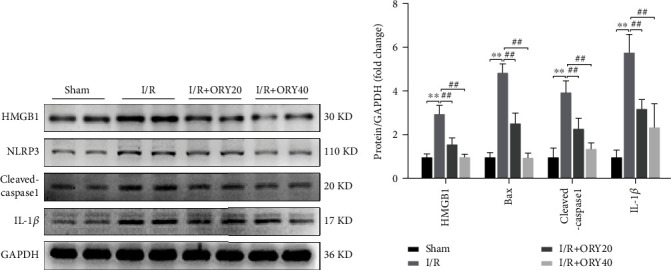
ORY attenuated HMGB1/NLRP3 inflammasome during HIRI. (a, b) Proteins of hepatic tissues were determined by western blotting for the determination of HMGB1, NLRP3, cleaved-caspase-1, IL-1*β*, and GAPDH expressions. Relative protein abundance was semiquantified by densitometry (*n* = 4). Data were expressed as mean ± standard deviation (SD) values. ^∗^*P* < 0.05, ^∗∗^*P* < 0.01, and ^∗∗∗^*P* < 0.001 versus the sham group; ^#^*P* < 0.05, ^##^*P* < 0.01, and ^###^*P* < 0.001 versus the I/R group; NS: no significance.

To investigate the protective effect of ORY against inflammation induced by I/R, we assessed the expression of HMGB1 and NLRP3 inflammasome-associated proteins, including NLRP3, cleaved-caspase-1, and IL-1*β* in the liver of the mice by western blotting. As shown in Figures 5(a) and 5(b), compared with the sham group, the protein abundance of HMGB1, NLRP3, cleaved-caspase-1, and IL-1*β* are significantly increased in the I/R group. In addition, ORY treatment significantly inhibited the activation of HMGB1, NLRP3, cleaved-caspase-1, and IL-1*β* in I/R-induced liver injury.

### 3.5. ORY Inhibited Cell Apoptosis during HIRI

**Figure 6:**
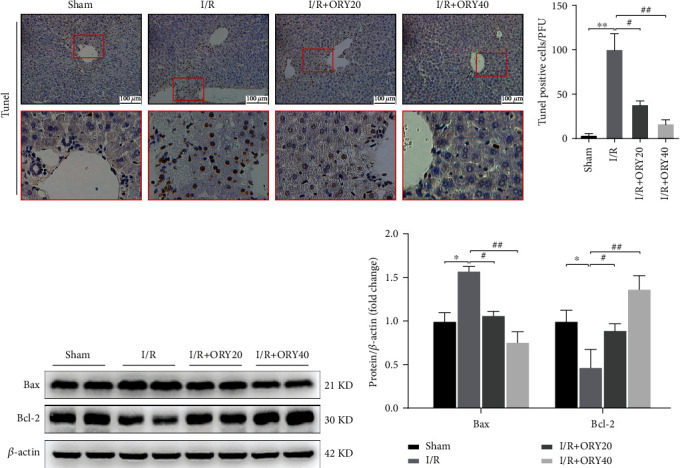
ORY inhibited cell apoptosis during HIRI. (a, b) TUNEL staining of hepacellular apoptosis (original magnification ×200). (c, d) Proteins of hepatic tissues were determined by western blot for the determination of Bcl-2, Bax, and *β*-actin expressions. Relative protein abundance was semiquantified by densitometry (*n* = 4). Data were expressed as mean ± standard deviation (SD) values. ^∗^*P* < 0.05, ^∗∗^*P* < 0.01, and ^∗∗∗^*P* < 0.001 versus the sham group; ^#^*P* < 0.05, ^##^*P* < 0.01, and ^###^*P* < 0.001 versus the I/R group; NS: no significance.

The number of apoptotic cells in mouse liver tissues was detected by TUNEL assay. As shown in Figures 6(a) and 6(b), there were only a few TUNEL-positive cells in the sham group, while plenty of cells in the I/R group. However, the number of TUNEL-positive cells in the ORY (20 and 40 mg/kg) pretreatment groups were significantly less than that in the I/R group (*P* < 0.01). Then, the protein expressions of apoptosis-related factors containing proapoptosis factor Bax and antiapoptosis factor Bcl-2 were measured via western blot in each group. It also showed that I/R significantly upregulated the expression of the proapoptotic protein Bax but downregulated the expression of the antiapoptotic protein Bcl-2, which were abolished by pretreatment with ORY (Figures 6(c) and 6(d)).

### 3.6. ORY Protects AML12 Cells from CoCl_2_-Induced Hypoxic Injury

**Figure 7:**
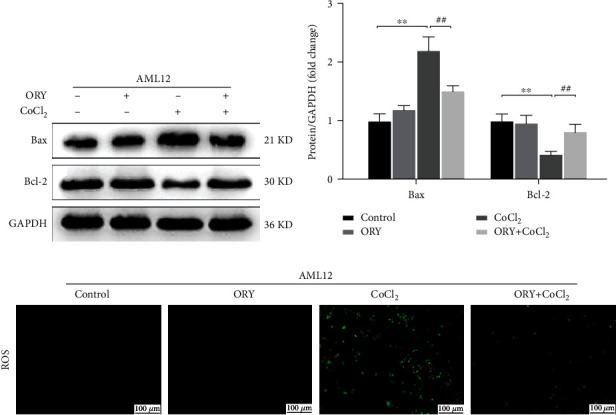
(a, b) Proteins of AML12 cells were determined by western blotting for the determination of Bax, Bcl-2, and GAPDH expressions. Relative protein abundance was semiquantified by densitometry (*n* = 3). Data were expressed as mean ± standard deviation (SD) values. (c) Cellular ROS estimated using the probe DCFH-DA by fluorescence microscopy. ^∗^*P* < 0.05, ^∗∗^*P* < 0.01, and ^∗∗∗^*P* < 0.001 versus the control group; ^#^*P* < 0.05, ^##^*P* < 0.01, and ^###^*P* < 0.001 versus the CoCl_2_ group.

In order to evaluate the effects of ORY against CoCl_2_-induced hypoxic injury, the AML12 cells were treated with 300 *μ*M CoCl_2_ and ORY (240 *μ*g/mL) for 24 hours. The proapoptosis factor Bax and antiapoptosis factor Bcl-2 were measured by western blot in each group. It also showed that CoCl_2_ significantly upregulated the expression of the proapoptotic protein Bax but downregulated the expression of the antiapoptotic protein Bcl-2, which were reversed by ORY (Figures 7(a) and 7(b)). The level of intracellular ROS can reflect the degree of cell injury induced by CoCl_2_. Compared with the CoCl_2_ group, ORY significantly reduced the levels of ROS (Figure 7(c)). These findings suggested that ORY protects AML12 cells from CoCl2-induced hypoxic injury.

### 3.7. ORY Suppressed AML12 Cells from CoCl_2_- or Tunicamycin-Induced Endoplasmic Reticulum Stress

**Figure 8:**
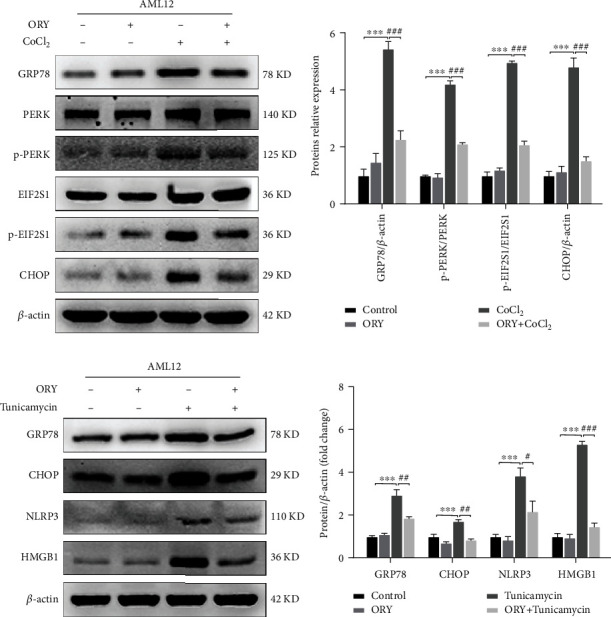
ORY suppressed AML12 cells from CoCl_2_- or tunicamycin-induced endoplasmic reticulum stress. (a, b) Western blotting analysis of GRP78, PERK, p-PERK, EIF2S1, p-EIF2S1, CHOP, and *β*-actin expressions in cells from the control, ORY, CoCl_2_, and CoCl2+ORY groups. (c, d) Western blotting analysis of GRP78, p-PERK, p-EIF2S1, CHOP, and *β*-actin expressions in cells from the control, ORY, tunicamycin, and tunicamycin+ORY groups. Relative protein abundance was semiquantified by densitometry (*n* = 3). Data were expressed as mean ± standard deviation (SD) values. ^∗^*P* < 0.05, ^∗∗^*P* < 0.01, and ^∗∗∗^*P* < 0.001 versus the control group; ^#^*P* < 0.05, ^##^*P* < 0.01, and ^###^*P* < 0.001 versus the CoCl_2_ group.

To investigate the protective effect of ORY on ERS-induced hypoxic injury in AML12 cells, we assessed the expression of GRP78, p-PERK, p-EIF2S1, and CHOP. As shown in Figures 8(a) and 8(b), CoCl_2_ significantly induced GRP78, p-PERK, p-EIF2S1, and CHOP, which were inhibited by ORY. As shown in Figures 8(c) and 8(d), AML12 cells were treated with tunicamycin (an ER stress activator) to induce ER stress. As shown in Figures 8(c) and 8(d), tunicamycin significantly induced GRP78, CHOP, NLRP3, and HMGB1, which were inhibited by ORY.

## 4. Discussion

HIRI is a clinically unavoidable destructive process that has a direct impact on liver tissue resection, trauma, hypovolemic shock, and transplantation [34]. However, according to statistics, 80% of liver transplant failure cases were accompanied with a high mortality rate after partial hepatectomy [35]. A plethora of dietary natural products relieved liver diseases and exerted protective effects on HIRI, which has the advantage of diversity of chemical structures, low cytotoxicity, high biological activity, and drug-like properties [36]. Rice contains numerous bioactive nutrients, including phenolics, anthocyanins, flavones, vitamin E, and *γ*-oryzanol (ORY), which plays an important role in maintaining health [37]. ORY, a dietary supplement in the United States, is known for its excellent antihyperlipidemic, anti-inflammatory, and antioxidant effect against liver diseases [30]. In this study, we used oral ORY supplementation as a preventive agent for I/R-mediated liver injury and investigated its underlying mechanism.

I/R injury is a multifactorial process that causes a series of serious clinical problems. In recent years, there have been numerous studies showing that oxidative stress, endoplasmic reticulum stress, and aseptic inflammation response-mediated liver injury make a momentous impact on HIRI. In normal physiological conditions, the antioxidative systems can maintain the redox balance of the body. During HIRI, overwhelming ROS accumulation could throw off that delicate balance, which therefore promotes cell apoptosis and tissue necrosis [38]. The SOD and GSH could be used to represent the degree of oxidative stress, due to their ROS scavenger properties [39]. However, MDA and MPO produced during oxidative stress can lead to liver tissue damage and antioxidant defense failure [40]. MPO activity is a marker of the amount of neutrophil. MDA is an indicator of lipid peroxidation. The results presented in the current study showed that ORY could decrease oxidative stress by reducing MDA and MPO content and elevating GSH and SOD content. This indicated that ORY has played an excellent antioxidation effect in HIRI.

Recently, studies have shown that oxidative stress can touch off ER stress by altering the structure and function of ER [41]. On the other hand, ER stress could exacerbate oxidative stress through ROS accumulation [42]. Corporately, two cellular stresses are often intertwined. ER, as an intracellular organelle, is responsible for protein synthesis and assemblage. Protein misfolding, accumulation of the aberrant proteins, and Ca^2+^ imbalance trigger the UPR which all lead to ER stress. The levels of GRP78, PERK, and CHOP, members of the UPR signaling pathway, were increased appreciably during HIRI. The results presented in the current study showed that HIR induced the upregulation of the levels of GRP78, p-PERK, p-EIF2S1, and CHOP. Oral ORY was able to significantly diminish the activation of ER stress. The same results were replicated at the cellular level. These results are in line with those of a recent study revealing that ORY directly ameliorates ER stress-induced *β*-cell dysfunction and subsequent apoptosis [43].

In addition, one commonly held view is that the aseptic inflammation response of hepatic cells leads to secondary liver damage. HMGB1, the prototypic DAMP molecule, plays a critical role at sterile inflammatory responses [44]. HMGB1 can combine exogenous (TLR2, TLR4, and TLR9) and endogenous (RAGE) ligands and subsequently induce activation of NLRP3 inflammasome and then active caspase-1 in the formation of mature IL-1*β* and IL-18 through ROS generation, which has a protective effect during the initial inflammation. Whereas when IL-1*β* and IL-18 are continually released and accumulated in the cell, they stimulate the endogenous inflammatory cascade, in turn, promote liver injury [45, 46]. Our study showed that the I/R group upregulated the protein expressions of HMGB1, NLRP3, caspase-1 (p20), and IL-1*β* compared with the sham group, and this result coincided with other studies [9, 47]. Furthermore, ORY pretreatment significantly reduced the protein expressions of HMGB1, NLRP3, caspase-1 (p20), and IL-1*β* compared with the I/R group. These results suggested that the protective effect of ORY on I/R-induced inflammatory responses might be associated with the suppression of the HMGB1/NLRP3/IL-1*β* signaling pathway.

Apoptosis, a form of programmed cell death, plays a crucial role in HIRI and manifests liver tissue damage directly. In this study, we demonstrated that the TUNEL-positive cells were dramatically increased in the I/R group. In addition, the mean number of TUNEL-positive cells in the ORY treatment groups was significantly less than that in the I/R group. Bcl-2 and Bax levels, which indicate the extent of apoptosis in the liver tissue, were analyzed in ischemic liver tissue after reperfusion. ORY treatment effectively repressed the upregulation of Bax levels and motivated the recovery of Bcl-2 levels after reperfusion, which were in line with the TUNEL results. Similarly, we found the same results in the CoCl_2_-induced hypoxia model and we used CoCl_2_ instead of an anoxic incubator mainly because the CoCl_2_-induced hypoxia model is more stable, and it has been used in other studies as well [48].

## 5. Conclusion

**Figure 9:**
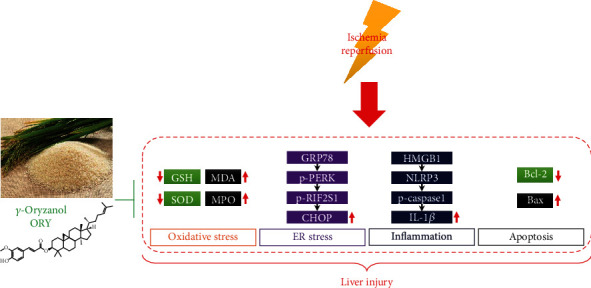
Putative model based on available data of protective effects of ORY during HIRI.

In summary, this study demonstrates that ORY ameliorated hepatic damage induced by I/R for the first time. Mechanically, ORY alleviated I/R-induced ER stress in the liver. Moreover, ORY pretreatment remarkably reduced the protein expressions of HMGB1, NLRP3, caspase-1 (p20), and IL-1*β* to protect the liver from I/R-induced inflammasome activation and apoptosis (Figure 9). Based on our data, ORY pretreatment would provide a new perspective for understanding the treatment and prevention of HIRI.

## Data Availability

Data used to support the findings of this study are available upon request.
